# Emotional Intelligence Not Only Can Make Us Feel Negative, but Can Provide Cognitive Resources to Regulate It Effectively: An fMRI Study

**DOI:** 10.3389/fpsyg.2022.866933

**Published:** 2022-06-10

**Authors:** Anita Deak, Barbara Bodrogi, Gergely Orsi, Gabor Perlaki, Tamas Bereczkei

**Affiliations:** ^1^Faculty of Humanities and Social Sciences, Institute of Psychology, University of Pécs, Pécs, Hungary; ^2^ELKH-PTE Clinical Neuroscience MR Research Group, Pécs, Hungary; ^3^Department of Neurology, Medical School, University of Pécs, Pécs, Hungary

**Keywords:** cognitive emotion regulation, emotional intelligence (EI), negative and neutral social stimuli, fMRI, emotional information processing

## Abstract

Neuroscientists have formulated the model of emotional intelligence (EI) based on brain imaging findings of individual differences in EI. The main objective of our study was to operationalize the advantage of high EI individuals in emotional information processing and regulation both at behavioral and neural levels of investigation. We used a self-report measure and a cognitive reappraisal task to demonstrate the role of EI in emotional perception and regulation. Participants saw pictures with negative or neutral captions and shifted (reappraised) from negative context to neutral while we registered brain activation. Behavioral results showed that higher EI participants reported more unpleasant emotions. The Utilization of emotions scores negatively correlated with the valence ratings and the subjective difficulty of reappraisal. In the negative condition, we found activation in hippocampus (HC), parahippocampal gyrus, cingulate cortex, insula and superior temporal lobe. In the neutral context, we found elevated activation in vision-related areas and HC. During reappraisal (negative-neutral) condition, we found activation in the medial frontal gyrus, temporal areas, vision-related regions and in cingulate gyrus. We conclude that higher EI is associated with intensive affective experiences even if emotions are unpleasant. Strong skills in utilizing emotions enable one not to repress negative feelings but to use them as source of information. High EI individuals use effective cognitive processes such as directing attention to relevant details; have advantages in allocation of cognitive resources, in conceptualization of emotional scenes and in building emotional memories; they use visual cues, imagination and executive functions to regulate negative emotions effectively.

## Introduction

Emotional intelligence (EI) consists of a set of social-emotional capacities (e.g., self-awareness, perceiving emotions, responding appropriately to emotions, regulating emotions) that can be either measured as a trait-like construct through self-report (Petrides et al., 2016) or as an ability of emotional reasoning and problem solving (Mayer et al., 2016). Despite the recent blooming literature on the role of EI in various behaviors from coping with COVID pandemia (Moroń and Biolik-Moroń, 2021) to academic performance (MacCann et al., 2020), the number of studies is limited about the neuropsychological basis of EI. The main goal of the current study is to contribute to a better understanding of the neural basis of processes related to the concept of EI.

According to the neurocognitive model of EI (Smith et al., 2018) individual differences can be defined at two main domains: (1) recognition and understanding emotions, (2) generating and regulating them. Neuroimaging studies report the interaction of several brain regions including the amygdala, the insular cortex, the ventromedial prefrontal cortex (vmPFC), and the anterior cingulate cortex (ACC).

In a social exchange reasoning task (Reis et al., 2007), higher ability EI correlated with hemodynamic responses in the left prefrontal cortex (BA10) and in the left anterior temporal brain areas (BA20). Both regions showed negative correlations with EI. Similarly, activation in the medial prefrontal cortex correlated negatively with trait EI while participants viewed static fearful faces (Killgore and Yurgelun-Todd, 2007). Ability (but not trait) EI correlated with elevated activation in the ventromedial PFC, amygdala, and insula in response to dynamically changing facial expressions indicative of trustworthiness (Killgore et al., 2013). In our study, we used 15 pairs of visual stimuli depicting social scenes, and we added a negative and a neutral caption to induce different affective atmospheres.

Structural volumetric studies also support the role of corticolimbic regions. Positive correlations were found between the EI scores and the volume of the insula, the medial PFC, and the anterior cingulate cortex (Killgore et al., 2012); positive associations with the insula, orbitofrontal cortex, parahippocampal gyrus, and negative correlation with the voxel-based morphometry measurements in the fusiform gyrus and middle temporal gyrus (Tan et al., 2014). Besides these, correlations were found in the key nodes of the social cognitive network and the default mode network (Takeuchi et al., 2011, 2013).

In the previous studies different EI scales have been used. Trait EI was measured by the Emotional Intelligence Scale (EIS) (Takeuchi et al., 2011), Trait Meta Mood Scale (TMMS) (Koven et al., 2011) and the Bar-On Emotional Quotient Inventory (EQ-i) (Killgore et al., 2012); ability EI was measured by the Mayer–Salovey–Caruso Emotional Intelligence Test (MSCEIT) (Killgore et al., 2012). To the best of our knowledge the Schutte Self-Report Emotional Intelligence Scale (SSREIS) was completed in only one gray matter volumetric study (Tan et al., 2014), thus our research is the first using the Hungarian version of SSREIS in a functional MRI study. The factor structure of the Schutte Self-Report Emotional Intelligence (SSREI) scale is sensitive to cultural differences. The original version (Schutte et al., 1998) measures global emotional intelligence with the 33 items in one factor. Petrides and Furnham (2000) identified four factors (1. Optimism/mood regulation; 2. Appraisal of emotions; 3. Social skills; 4. Utilization of emotions), but the Chinese version used by Tan et al. (2014) and Li et al. (2021) neuroimaging study has a different structure. Due to cultural and methodological reasons they removed items that measure optimism and appraisal of emotions in self. Thus, the Chinese version has also a four-factor structure but a little bit different from the western one (1. Monitor of Emotions; 2. Utilization of Emotions; 3. Social ability; 4. Appraisal of Emotions in others). The authors of the Hungarian version (Kun et al., 2010) tested different models (one-, three-, four-, six-factor solutions), and the three-factor structure proved to be the most convincing (see Measures).

In the present study, we used fMRI to register brain activation in a cognitive emotion regulation (CER) task. In contrast to previous studies that applied a research design with facial stimuli (Killgore and Yurgelun-Todd, 2007; Killgore et al., 2012), or a social decision task, our CER task is unique because it enables us to evaluate the relationship between neural response and EI in three aspects to provide a global picture of emotional information processing: (1) during watching a visual stimulus with negative caption; (2) during watching a visual stimulus with neutral caption; and (3) shifting from one context to another (e.g., from the negative to the neutral) (reappraisal). Based on the neurocognitive model of EI (Smith et al., 2018) we hypothesize that emotional intelligence as measured by self-report correlate with the neural response of the following regions: amygdala and insula in the negative condition, anterior cingular cortex and PFC in the reappraisal condition.

## Materials and Methods

### Participants

Fourty right-handed young adults participated in the experiment (mean age = 21.03 years, SD = 2.1; 19 men). None of them reported any neurological history. All participants gave written informed consent before scanning. The study was approved by the United Ethical Review Committee for Research in Psychology (EPKEB; reference number 2017/126). Procedures were in accordance with the 1964 Declaration of Helsinki.

### Measures

#### Emotional Intelligence (Assessing Emotions Scale)

Participants completed the Hungarian version (Kun et al., 2010) of the 33- item questionnaire of Schutte et al. (1998) (Assessing Emotions Scale [AES-HU]). Different models were tested (one-, three-, four-, six-factor solutions), and the three-factor structure proved to be the most convincing based on the Hungarian data. The first factor, ‘Appraisal of Emotions’, refers to the appraisal of signs and messages concerning one’s own or others’ emotions. The second factor is the ‘Optimism and Emotion Regulation’. It measures how one evaluates his own abilities of changing and regulating emotions. The third factor, ‘Intrapersonal and Interpersonal Utilization of Emotions’, refers to how the person perceives and to what extent he can use his mood changes in cognitive processes. (Cronbach’s alpha values for the current sample: Total score: 0.88; Appraisal of Emotions: 0.78; Optimism and Emotion Regulation: 0.69; Intrapersonal and Interpersonal Utilization of Emotions:0.86.)

#### Cognitive Emotion Regulation Task

The Cognitive Emotion Regulation (CER) task was identical with Bodrogi et al. (2020). Social-emotional scenarios were selected from a standard affective stimulus database (Lang et al., 2005; Deak et al., 2010) and two different captions, a negative and a neutral were added to each picture based on results of previous pilot studies. Altogether 15 pairs of pictures were presented one by one. The two different captions of each picture were displayed one after the other. Participants were instructed to have a look at the picture (e.g., with the negative caption) and let their emotions flow in response to the stimulus. Then the same picture appeared with the other caption (e.g., with the neutral). The new caption would allow the participants to reframe the situation from another point of view (i.e., less unpleasant with the neutral caption than with the negative caption). For example, the picture of an old man standing by the window had a negative caption “Goodbye”, and “Waiting (for the grandkids)” as a neutral caption. By attaching the same visual stimulus to two distinctive contexts (negative vs. neutral), participants had to perform reappraisal as a cognitive emotion regulation strategy. Outside the scanner, participants evaluated all images one by one with both captions regarding valence and arousal; they also rated how difficult the reappraisal had been (see Supplementary Material). As an index of emotion regulation success, we have calculated differential scores both for valence and arousal (neutral-negative ratings) (i.e., Valence difference, Arousal difference).

### Procedure

Before scanning, participants were told the instruction while they saw a pair of trial pictures. A trained research assistant explained the task and registered the answers until the task was fully understood by the participant. After scanning, the participants were presented with the same stimuli as inside the scanner and we asked the following questions: (A) context for the first picture (participants briefly described the scene with negative title), (B) emotions (participants freely listed associated emotions), (C) valence (participants used a 9-point Likert scale where 1 = unpleasant and 9 = pleasant feelings), (D) arousal (participants used a 9-point Likert scale where 1 = low arousal and 9 = high arousal). The same A-D answers were collected for the pictures with neutral titles, before participants gave a subjective difficulty rating on a 9-point Likert scale. 1 indicated an easy way for shifting from one context to another, 9 represented strong difficulties of interpreting the picture from the second point of view, so the switch between the two meanings was complicated (see Supplementary Material).

### Functional MRI Data Acquisition and Analysis

MRI data were acquired with a 3-T scanner (MAGNETOM Trio, Siemens AG, Erlangen, Germany) at the Diagnostic Center of Pecs (Pecs, Hungary) equipped with a 12-channel head coil. Functional MRI was based on a gradient-echo echo planar imaging sequence (TR = 2000 mm, TE = 36 ms, flip angle = 76°, FOV = 230 mm × 230 mm, matrix = 92 × 92, ST = 4 mm, no gap) with a spatial resolution of 2.5 × 2.5 × 2.5 mm (23 slices with interleaved slice order) in an axial orientation parallel to the AC--PC plane. Motion correction was according to the manufacturer’s instructions. Image processing was carried out using SPM12^1^ implemented in MATLAB (Version 9.11.0.187 [R2021b] Service Pack 1) (Mathworks Inc., Sherborn, MA). Images were corrected for motion, registered to the standard space at 2 × 2 × 2 mm, normalized to the SPM12 template, and smoothed with a full-width-at-half-maximum Gaussian kernel of 5 mm.

We computed a first-level, subject-wise analysis using a general linear model with a hemodynamic response function modeling the emotional information processing. The following regressors were used to define the design matrix: (1) NEGATIVE condition, (2) NEUTRAL condition, (3) BASELINE (“relax” instruction), and (4) CONTROL (the scrambled version for each image). (We do not use this latter one while we report the results.) We defined the Negative > Baseline, the Neutral > Baseline, Negative > Neutral and Neutral > Negative contrasts for each participant, and a first-level analysis was computed by modeling the information processing of negative stimuli and neutral stimuli, respectively. The Negative > Neutral and Neutral > Negative contrasts were used to model reappraisal, that is shifting from the negative to the neutral context and vice versa. As a second-level analysis, we used one-sample t test for the group activity during the information processing of negative and neutral stimuli, respectively, as well as during the shift from negative to neutral and neutral to negative captions. EI Total scores were added as a regressor.

## Results

The EI Total score negatively correlated with the valence ratings for pictures with the negative caption (*r* = −0.31 *p* < 0.05) (see Table 1 and Supplementary Material). It means individuals with higher EI rated the pictures more unpleasant and reported less difficulties during the reappraisal task. All subscales of AES-HU correlated with the valence difference (AE: *r* = 0.44, *p* < 0.05; OER: *r* = 0.37, *p* < 0.05; UE: *r* = 0.29, *p* < 0.07), that is stronger skills in each component of EI are related to bigger affective distance one has while using reappraisal as a cognitive emotion regulation strategy. We found a marginal positive correlation between the AE subscale score and the arousal rating of pictures with negative captions (*r* = 0.31, *p* < 0.06); it means that individuals with stronger skills in the appraisal of emotions give higher arousal ratings (i.e., more intensive emotions in response to pictures with negative captions). The UE subscale score negatively correlated with the valence rating of pictures with negative captions (*r* = −0.37, *p* < 0.05), and with the subjective task difficulty (*r* = −0.32, *p* < 0.05). It indicates that stronger skills in the utilization of emotions are associated with lower valence ratings (i.e., more unpleasant emotional response to pictures with negative captions) and less subjective level of emotion regulat difficulty while individuals shift from the negative to the neutral context.

**TABLE 1:** Means (M), standard deviations (SD) and correlations between self-report EI (AES Total score and subscales) and task-specific variables (*N* = 40).

	AES Total	AE	OER	UE	M (SD)
Negative valence	−0.31*	−0.18	−0.16	−0.37*	3.59 (0.67)
Negative arousal	0.23	0.31^#^	0.13	0.11	6.91 (1.03)
Neutral valence	0.18	0.23	0.25	−0.03	5.41 (0.72)
Neutral arousal	0.21	0.19	0.06	0.18	6.41 (1.08)
Valence difference	0.44**	0.37*	0.37*	0.29^#^	1.82 (0.77)
Arousal difference	0.02	−0.19	−0.10	0.13	0.50 (0.57)
Subjective task difficulty	−0.24	−0.17	0.02	−0.32*	3.89 (1.24)
M	104.23	32.25	19.05	26.53	
(SD)	(14.64)	(5.62)	(3.32)	(5.21)	

*p < 0.05; ^#^p = 0.07; AE = Appraisal of Emotions; OER = Optimism and Emotion Regulation; UE = Intra-and Interpersonal Utilization of Emotions.

**FIGURE 1:**
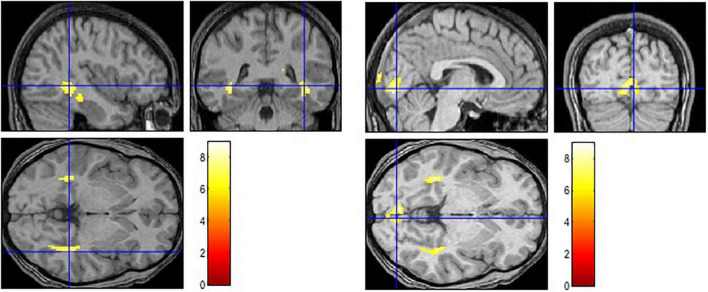
Activation in limbic and paralimbic regions in the negative condition (Negative > Baseline) (left) and in higher-order vision-related regions in the neutral condition (Neutral > Baseline) (right).

The contrast examining the brain activation in response to the picture with negative caption (Negative > Baseline) yielded bilateral response in the hippocampus, parahippocampal gyrus and posterior cingulate cortex (see Figure 1 and Table 2). Moreover, significant activation was found in the left superior temporal pole and in the insula. When the neutral condition was contrasted to the baseline (Neutral > Baseline), increased activation was detected in the bilateral calcarine, lingual gyrus, cuneus, hippocampus and parahippocampal gyrus. The Negative > Neutral contrast yielded bilateral activation in the superior medial frontal gyrus, superior and middle temporal gyrus. Furthermore, we found significant neural response in the left cuneus, precuneus, calcarine, angular gyrus, lingual gyrus, middle occipital gyrus, and in the right posterior and middle parts of the cingulate cortex, as well as in the right inferior temporal gyrus. For the Neutral > Negative contrast, we found elevated bilateral activation in the middle and superior occipital gyri, cuneus, lingual gyrus, calcarine, superior parietal lobe, cerebellum, hippocampus, and thalamus, and in the right angular gyrus.

**TABLE 2:** Brain regions in response to pictures with negative caption (Negative > Baseline), neutral caption (Neutral > Baseline) and reappraisal (Negative > Neutral; Neutral > Negative) while using participants’ EI Total score as a regressor.

Brain region	Voxels	MNI coordinates			t-value
		x	y	z	
**Negative > Baseline**					
R Hippocampus/Parahippocampal gyrus	376	24	–46	18	8.96
L Hippocampus/Parahippocampal gyrus	137	–36	–44	–4	8.57
R Posterior cingulate	18	14	–36	8	8.27
L Posterior cingulate	26	–6	–34	12	7.06
L Superior Temporal pole	9	–42	20	–10	6.28
L Insula	5	–44	16	4	6.27
**Neutral > Baseline**					
R Cuneus, Calcarine, Lingual gyrus	189	8	–102	12	8.97
L Lingual gyrus, Calcarine	75	–4	–76	–4	8.67
L Cuneus, Superior occipital gyrus	24	–8	–104	14	7.50
L Hippocampus/Parahippocampal gyrus	78	–38	–40	–4	8.16
R Hippocampus/Parahippocampal gyrus	109	38	–42	–10	7.77
**Negative > Neutral**					
L Cuneus/Precuneus/Calcarine	925	–6	–70	–18	13.82
L Lingual gyrus	209	–16	–50	–2	12.83
R Posterior and middle cingulate gyrus	532	20	–38	14	6.92
L Middle occipital gyrus, Angular gyrus	177	–46	–64	18	11.22
L Superior and middle temporal gyri	329	–54	–66	20	11.03
R Superior temporal gyrus	557	52	–16	–24	6.64
R Middle and inferior temporal gyri	327	54	–62	4	11.50
R Superior Medial Frontal gyrus	19	8	54	30	6.24
L Superior Medial Frontal gyrus	5	–4	52	30	6.09
**Neutral > Negative**					
L Middle and superior occipital gyri, Cuneus	1450	–6	–80	–8	20.45
R Middle and Superior occipital gyri, Cuneus	1213	10	–88	–12	20.45
L Lingual gyrus/Calcarine	547	–6	–80	–8	20.45
R Lingual gyrus/Calcarine	1293	4	–84	2	20.45
L Cerebellum	24	–6	–80	–8	20.45
R Cerebellum	60	6	–72	–4	20.45
L Superior Parietal Lobe	529	–6	–80	–8	20.45
L Hippocampus/Thalamus	27/22	–22	–30	–4	9.68
R Hippocampus/Thalamus	16/6	22	–28	–4	7.29
R Angular Gyrus/Superior Parietal Lobe	84/12	26	–64	50	7.38

*L = Left; R = Right; FWE corrected, p < 0,05, k > 5.*

## Discussion

In this study we used the concept of EI to identify individual differences in emotional information processing and emotion regulation both at behavioral and neural levels of investigation. We applied a cognitive emotion regulation task that consisted of two phases: (1) participants saw a social scene with a caption inducing a negative emotional atmosphere and (2) the same visual stimulus with a neutral caption providing a different, non-emotional interpretation. Inside the scanner, participants were instructed to let their feelings flow in response to the stimuli. Outside the scanner they rated the pleasantness and the arousal of each picture with both captions (negative and neutral), as well as they reported how difficult it was to shift between the two images. This subjective difficulty rating served as an indicator of task-specific emotion regulation. Additional indicators for valence and arousal, respectively, were computed to measure the success of emotion regulation. Valence and arousal difference refer to the affective distance that individuals had between the negative and the neutral contexts.

The negative correlations between the valence ratings of pictures with negative captions and EI Total score, as well as with the utilization of emotion component of EI, support previous behavioral findings showing that a high level of EI contributes to the evaluation of the valence of the emotional stimulus and to experience more unpleasant feelings (Peña-Sarrionandia et al., 2015; Bodrogi et al., 2020).

Additional significant correlations between the components of trait EI (i.e., utilization, appraisal, optimism and emotion regulation), and task-specific indicators of emotion regulation (i.e., valence difference, subjective task difficulty) demonstrate the existing relationship between EI and ER (Megías-Robles et al., 2019). We identified that the ability to utilize emotions plays a specific contribution to ER. The stronger skills one has in utilizing emotions for intra-and interpersonal purposes the more negative emotions and the less difficulty he experiences in regulating them through cognitive strategies. Although emotional utilization consists of processes involved in making adaptive use of arousal (Izard et al., 2011), our results point out that the constructive usage of emotional information is not limited to arousal. We conclude that valence ratings and valence differences between negative and neutral conditions proved to be important, namely, high EI skills (regarding general EI and specifically the emotional utilization as an EI component) are in association with the experience of unpleasant emotions. Moreover, the emotional distance (e.g., the valence difference) one has while uses a cognitive emotion regulation strategy such as reappraisal, is a key indicator that corresponds with general EI and its components (i.e., appraisal, emotion regulation and utilization).

We used EI Total score as a covariate while we calculated the brain activation maps in response to pictures with negative and neutral captions, respectively. Limbic and paralimbic regions (hippocampus, parahippocampal gyrus, insula, cingulate) played a crucial role in the negative condition, like in former studies (e.g., Killgore and Yurgelun-Todd, 2007). As part of the social cognitive network (Takeuchi et al., 2013), the activation of these regions may reflect an interaction between emotion and memory, such as the enhancement of memory for social-emotional information (Pearson et al., 2011). Our findings also support the role of insula as a key node in the emotional intelligence circuitry (Killgore et al., 2012). Additionally, the insula serves as the part of a salience network (Smith et al., 2018), which directs attention to emotionally relevant stimuli.

We found activation in the bilateral hippocampus and parahippocampal gyrus not only in the negative but in the neutral condition, as well. This result points out that they play an important role in assigning conceptual meaning to the current percepts in a context-sensitive manner (Smith et al., 2018). In response to the pictures with neutral caption, we found activation in vision-related areas (e.g., lingual gyrus, calcarine, superior occipital gyrus, cuneus). This result underlined previous studies that higher EI scores are associated with significantly greater signal change within the occipital lobe (Killgore and Yurgelun-Todd, 2007). Our CER task demanded to have increased attention to one’s own intrapersonal affective response to the stimuli and to have strong skills in identifying and understanding affective signals in a social scene. It might be the case that high EI participants followed our instruction successfully to imagine the situation, and the calcarine activation refers to their visual-mental imagery (Klein et al., 2000). Activation in the lingual gyrus also supports that high EI individuals may have an increased processing of emotional information at higher level visual areas (Nomi et al., 2008; Pattyn et al., 2021). From the brain activation in the Neutral > Negative contrast we conclude that an intensive visual mapping occurred involving not only the vision-related areas (e.g., middle and superior occipital gyri, lingual gyrus, cuneus, calcarine) but the superior parietal lobe (SPL). The SPL was found active in previous studies where sustained attention was directed to emotionally neutral stimuli (Le et al., 1998 in Beauregard et al., 2001) or emotion regulation processes were required (Morawetz et al., 2017; Pozzi et al., 2021). In line with recent meta-analyses (Morawetz et al., 2017; Pozzi et al., 2021), our brain imaging results of reappraisal (Negative > Neutral) pointed out the role of medial prefrontal cortex, the cingulate gyrus and temporal cortical areas.

Based on the limbic and paralimbic neural response we conclude that higher EI is associated with deeper affective experiences even if emotions are unpleasant. High EI individuals may use effective cognitive processes such as directing attention to emotionally relevant details of a context, and they may have advantages in allocation of cognitive resources, in conceptualization of emotional scenes and in building emotional memories. Previous findings demonstrated the moderating role of trait EI on memory and attention (Mikolajczak et al., 2009) indicating that the regulatory components of EI focused attention to neutral material and facilitated memory for positive events in a neutral condition. However, in the negative condition high EI individuals engaged attention to the emotional material and recalled more negative details, while low EI individuals disengaged attention from emotional material and recalled more positive events.

Moreover, they may have increased emotional information processing including strong skills to utilize visual affective cues, and they use their imagination enriched with mental representations to regulate their negative emotions effectively. We conclude that our study supports the connection between EI and the activation of the social-cognitive network including the medial prefrontal cortex, precuneus, cingulate cortex and superior temporal areas (Takeuchi et al., 2013).

Based on preceding literature, we expected amygdala activation in the negative condition (during viewing a picture with negative caption). Our results, however, showed no amygdala response. The lack of amygdala activation might indicate that the social scenes we selected as stimuli were neither unpleasant enough or highly arousing to alarm the amygdala.

Besides the conceptual and methodological uniqueness of our study that it is the first using the Hungarian version of SSREIS in a functional MRI study while participants perform a CER task, there are limitations. For example, the EI Total scores were added to the fMRI analysis. It is a next step to use the subscales of AES-HU not only in the behavioral but also in the neuroimaging data analysis. As participants rated the pictures outside the scanner, a possible repetition effect was present. Further studies should clarify the impact of repeated exposure on task-specific variables (e.g., valence and arousal ratings, subjective level of difficulty). A possible future direction of studies could investigate the impact and/or the effectiveness of an EI training to follow the changes at both psychological and neuropsychological levels.

## Data Availability Statement

The raw data supporting the conclusions of this article will be made available by the authors, without undue reservation.

## Ethics Statement

The studies involving human participants were reviewed and approved by United Ethical Review Committee for Research in Psychology (EPKEB) Reference number: 2017/126. The participants provided their written informed consent to participate in this study.

## Author Contributions

AD: conceptualization, research design development, data analysis, and manuscript writing. BB: data collection, data analysis, and manuscript writing. GO and GP: research design development and fMRI data collection. TB: conceptualization, interpretation of results, and manuscript review. All authors contributed to the article and approved the submitted version.

## Conflict of Interest

The authors declare that the research was conducted in the absence of any commercial or financial relationships that could be construed as a potential conflict of interest.

## Publisher’s Note

All claims expressed in this article are solely those of the authors and do not necessarily represent those of their affiliated organizations, or those of the publisher, the editors and the reviewers. Any product that may be evaluated in this article, or claim that may be made by its manufacturer, is not guaranteed or endorsed by the publisher.

## Footnotes

1
www.fil.ion.ucl.ac.uk/spm
