# Supplementary material for: Emotional Intelligence Not Only Can Make Us Feel Negative, but Can Provide Cognitive Resources to Regulate It Effectively: An fMRI Study
Source: Front Psychol. 2022 Jun 10;13:866933. doi: 10.3389/fpsyg.2022.866933 (PMC9226432; doi:10.3389/fpsyg.2022.866933)
Supplement: Supplementary file 1 [file Data_Sheet_1.docx]

***Supplementary Material***


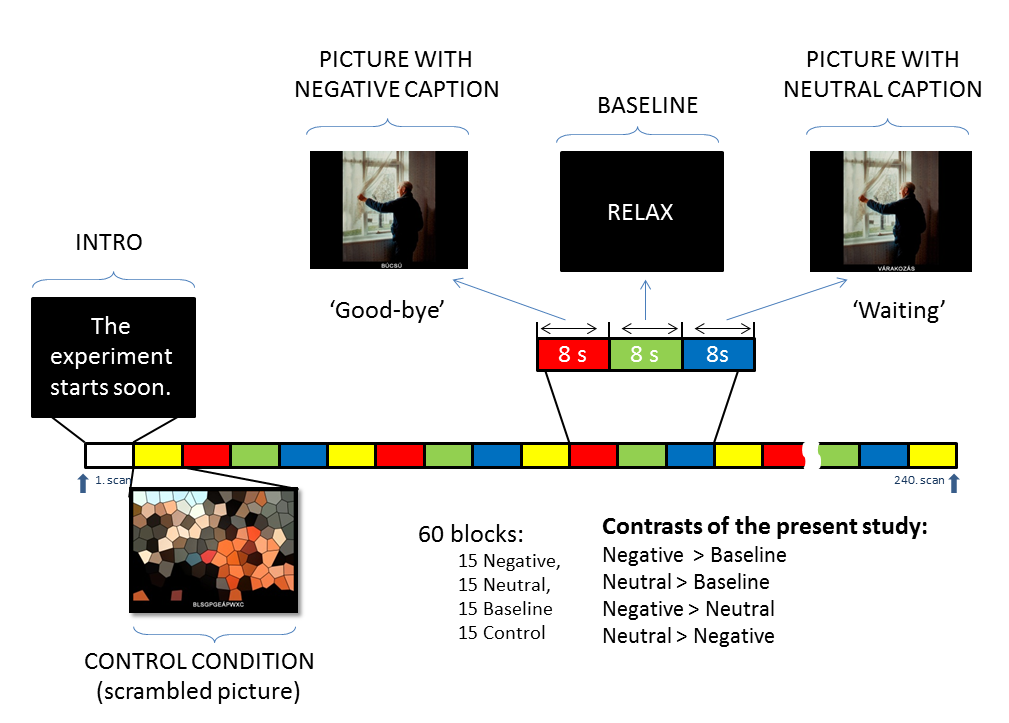


**Supplementary Figure 1.** Experimental design.


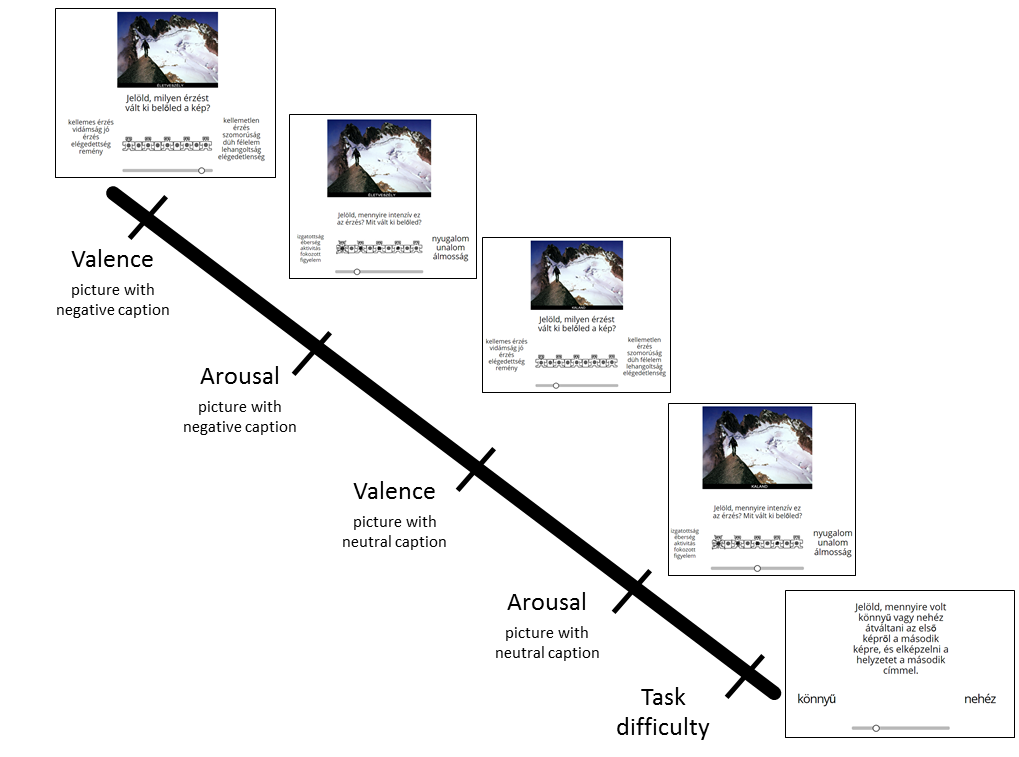


**Supplementary Figure 2.** Sequence of data acquisition for post-scan ratings

|  | With negative caption | | With neutral caption | |
| --- | --- | --- | --- | --- |
|  | valence | arousal | valence | arousal |
| 2480 | 3,58 | 6,15 | 7,05 | 6,43 |
| 2616 | 3,65 | 5,95 | 6,95 | 6,33 |
| 2691 | 3,93 | 6,68 | 4,87 | 7,08 |
| 4598 | 2,33 | 7,53 | 8,28 | 7,95 |
| 5629 | 3,00 | 7,13 | 7,93 | 7,28 |
| 6212 | 1,48 | 7,95 | 6,10 | 7,08 |
| 6250 | 2,41 | 7,51 | 5,23 | 5,43 |
| 6360 | 2,33 | 7,13 | 5,25 | 5,00 |
| 6530 | 1,95 | 6,95 | 6,90 | 5,70 |
| 6825 | 2,53 | 6,15 | 5,50 | 6,10 |
| 6836 | 3,93 | 6,63 | 5,98 | 5,90 |
| 8010 | 2,58 | 6,18 | 7,93 | 6,78 |
| 9050 | 2,03 | 7,03 | 5,03 | 6,55 |
| 9102 | 3,05 | 6,93 | 4,28 | 5,78 |
| 9421 | 2,08 | 7,30 | 6,83 | 7,20 |

**Supplementary Table 1.** Valence and arousal mean ratings (N=40) for pictures with negative and neutral captions, respectively.
